# Oncologist uptake of comprehensive genomic profile guided targeted therapy

**DOI:** 10.18632/oncotarget.27047

**Published:** 2019-07-23

**Authors:** Mary K. Nesline, Paul DePietro, Grace K. Dy, Amy Early, Antonios Papanicolau-Sengos, Jeffrey M. Conroy, Felicia L. Lenzo, Sean T. Glenn, Hongbin Chen, Anne Grand’Maison, Patrick Boland, Marc S. Ernstoff, Igor Puzanov, Stephen Edge, Stacey Akers, Mateusz Opyrchal, Gurkamal Chatta, Kunle Odunsi, Peter Frederick, Shashikant Lele, Mark Gardner, Carl Morrison

**Affiliations:** ^1^OmniSeq Inc., Buffalo, NY 14203, USA; ^2^Department of Medicine, Roswell Park Comprehensive Cancer Center, Buffalo, NY 14263, USA; ^3^Department of Pathology, Roswell Park Comprehensive Cancer Center, Buffalo, NY 14263, USA; ^4^Center for Personalized Medicine, Roswell Park Comprehensive Cancer Center, Buffalo, NY 14263, USA; ^5^Cancer Genetics and Genomics, Roswell Park Comprehensive Cancer Center, Buffalo, NY 14263, USA; ^6^Division of Gynecologic Oncology, Roswell Park Comprehensive Cancer Center, Buffalo, NY 14263, USA; ^7^Department of Surgery, Roswell Park Comprehensive Cancer Center, Buffalo, NY 14263, USA

**Keywords:** comprehensive genomic profiling, targeted therapy, clinical decision making, next-generation sequencing, real world data

## Abstract

We describe the extent to which comprehensive genomic profiling (CGP) results were used by oncologists to guide targeted therapy selection in a cohort of solid tumor patients tested as part of standard care at Roswell Park Comprehensive Cancer Center June 2016–June 2017, with adequate follow up through September 2018 (*n =* 620). Overall, 28.4% of CGP tests advised physicians about targeted therapy use supported by companion diagnostic or practice guideline evidence. Post-test targeted therapy uptake was highest for patients in active treatment at the time of order (86% versus 76% of treatment naïve patients), but also took longer to initiate (median 50 days versus 7 days for treatment naïve patients), with few patients (2.6%) receiving targeted agents prior to testing. 100% of patients with resistance variants did not receive targeted agents. Treatment naïve patients received immunotherapy as the most common alternative. When targeted therapy given off-label or in a trial was the best CGP option, (7%) of patients received it. Our data illustrate the appropriate and heterogeneous use of CGP by oncologists as a longitudinal treatment decision tool based on patient history and treatment needs, and that some patients may benefit from testing prior to initiation of other standard treatments.

## INTRODUCTION

Despite growing routine use by oncologists, the usefulness of comprehensive genomic profiling (CGP) by next generation sequencing (NGS) for targeted therapy treatment decisions in real world clinical practice has gone largely uncharacterized. Response rates and outcomes for variant-directed targeted therapy are superior to non-biomarker based therapy as demonstrated in the literature of retrospective meta-analyses of clinical trials in advanced and metastatic cancer patients [1–3]. Comprehensive genomic profiling (CGP) supports putting these results into practice by leveraging the high-throughput capabilities of next generation sequencing (NGS) technologies to simultaneously test for all types of genomic alterations, including mutations, copy number variants (amplification) and fusions (rearrangements), associated with targeted therapy opportunities for a panel of cancer associated genes. GCP intends to replace one-at-time, drug and tumor-type specific companion diagnostic tests that are designed to detect only a small subset of the potential actionable alterations for one variant type in a single gene. For example, fluorescent *in situ* hybridization (FISH) is the gold standard for measuring rearrangements in multiple genes such as *ALK, RET, ROS1*, and *NTRK*, which are highly predictive of response to tyrosine kinase inhibitors (TKIs) such as alectinib, cabozantinib, ceritinib, crizotinib, and larotrectinib. However, FISH testing for these genes, unlike RNA-based NGS testing, has been shown to be at risk for false positives [4], and may under-report actionable alterations that could result in denying patients treatment with these highly efficacious TKI inhibitors [5, 6]. Similarly, CGP can detect highly actionable *EGFR* mutations that predict response to EGFR inhibitors in NSCLC that have been missed by single gene testing [7].

The prevalence and actionability of variants for targeted therapy treatment decision-making also vary widely by histology. BRAF V600E mutations for example, required for selection of combination BRAF/MEK inhibitors, occur in one-third to one-half of melanomas [8], but only in 2–4% of non-small cell lung cancer (NSCLC) patients [9], both of which are on-label FDA approved indications for BRAF/MEK inhibitors. In colorectal cancer, BRAF V600E mutations occur in about 10% of patients [10] and indicate non-response to anti-EGFR therapy with cetuximab or panitumumab as first line treatment for metastatic disease [11], whereas triplet encorafenib + binimetinib + cetuximab was granted FDA breakthrough designation for BRAF V600E colorectal cancer in the second line setting based on results from the phase III BEACON trial [12]. The heterogeneous application of results for the same alteration within and across multiple tumor types underscores the clinical relevance of concurrently examining a broad range of genomic alterations by CGP.

Payers have potentially overlooked CGP as a sophisticated testing approach that informs complex clinical decision-making and enables a patient’s next best care option, be it drug therapy or no further treatment. A few studies have assessed the benefits of CGP for treatment decisions, but results often have limited generalizability with patients tested in the context of prospective studies intended to enroll patients in targeted therapy trials [13–18], limited histologies [19], using retrospective physician questionnaires with modest response rates at the patient level [20, 21], or most commonly, in the context of large academic center molecular tumor boards [22–26].

CGP endeavors to be a standard of care pan-cancer tool that tests for all major variant classes associated with potential targeted therapy options and contraindications, including agents with on-label approvals, supported by professional practice guidelines, or off-label and investigational agents, spanning multiple treatment settings. Given the broad scope of potential CGP applications, understanding oncologists’ intended use of results for first or subsequent line therapy selection, and assessment of post-test therapeutic interventions, are both needed to determine usefulness of CGP as a treatment decision making tool. This is a challenge for reference laboratories that perform CGP because they receive very limited clinical context about patients at the time of test order, and must depend on collaboration with oncology treatment centers to provide subsequent clinical and treatment data for tested patients to demonstrate test utility. While government and private payers require CGP be medically necessary for patient treatment to be covered as a service under health plans, labs are very limited in accessing clinical documentation that details patient need for testing.

The objective of this study therefore, was to address current limitations in understanding CGP utility by describing post-test treatment decisions, accounting for prior patient treatment history and the actionability of results, in a cohort of advanced/metastatic solid tumor patients. Specifically, we classified all treatments (targeted therapy, immunotherapy, chemotherapy and other standard of care treatments, clinical trials, or no treatment), and when they were received (pre- or post-test) from electronic medical records as of September 2018, for CGP tests consecutively performed for Roswell Park Comprehensive Cancer Center patients between June 2016 and June 2017.

CGP testing was completed in a CLIA certified laboratory (OmniSeq, Inc., Buffalo, NY, USA), using OmniSeq Comprehensive^®^, a commercially available test approved for clinical use by the New York State Clinical Laboratory Evaluation Program (NYS CLEP). The OmniSeq Comprehensive CGP assay uses DNA sequencing of tumor tissue to identify somatic alterations in 144 cancer-associated genes, including single nucleotide variants, insertions, deletions, indels, and copy number variants, and RNA sequencing to perform rearrangement (fusion) analysis in oncogenes. CGP test results were classified by the level of evidence supporting the targeted therapy guidance provided in final reports as: level 1 companion diagnostic; level 2 practice guidelines; or level 3 off-label/clinical trials [27].

We used patient treatment history status on the day of test order (treatment naïve, in active treatment, or previously treated at least 120 days prior) as a proxy indicator for intended use of results (i.e., to guide first line treatment versus identifying next potential therapy). We then compared all administered post-test treatment(s) to the treatment guidance provided by each CGP report, classified by evidence groups, to gauge the extent to which physicians used CGP results to make treatment decisions overall. We hypothesized that oncologist uptake of CGP-guided targeted therapy, where recommended, would be mediated by patient treatment history status at the time of test order.

## RESULTS

### Test and patient characteristics

**Figure 1:**
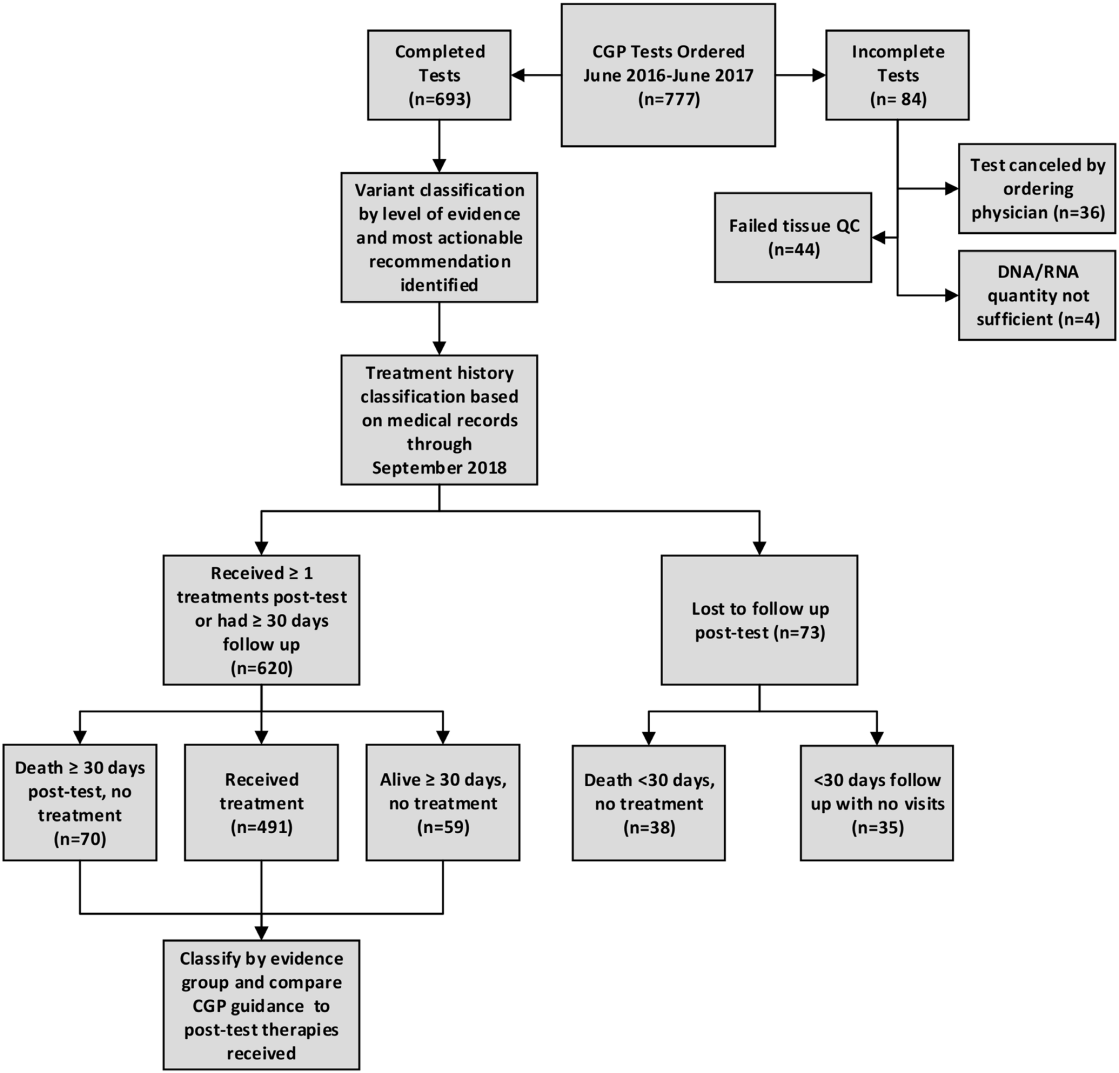
Study Schema describing process for CGP test inclusion, classification of results, assessment of pre-and post-test treatment changes, and uptake of CGP treatment recommendations.

For 777 test orders between June 2016 and June 2017 at Roswell Park Comprehensive Cancer Center (Figure 1), 84 (10.8%) were excluded from analysis because they were never completed, due to physician cancelation (36/777; 4.6%), failed up front tissue quality control (44; 5.7%), or the DNA/RNA quantity was not sufficient for testing (4/777; <1%). After medical record review, an additional 73 tests (9.4%) were excluded as lost to follow up due to death within 30 days of CGP results with no subsequent therapy (*n* = 38; 4.9%), or to having less than 30 days follow up time following report delivery with no subsequent visits to Roswell Park (*n* = 35; 4.5%). We included 620 (79.8%) tests that were both completed (resulted) to physicians, and that had adequate follow up documentation available from medical records in analysis, with 491/620 (79%) either receiving at least one therapy by the end of the observation period, or accruing at least 30 days of follow up with no further treatment and/or death by the end of the observation period (129/620; 21%).

**Figure 2:**
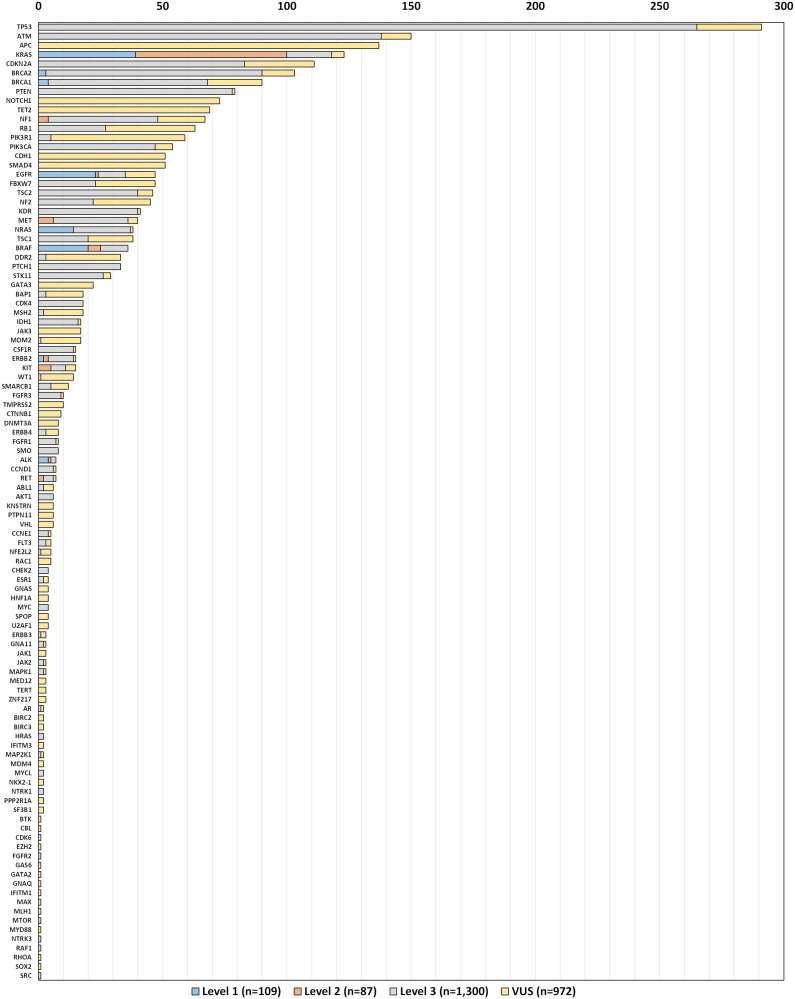
Number of variants detected by gene and level of evidence (*n* = 620).

For the 620 included tests, a total of 2,468 variants were detected in 104 genes (Figure 2). It should be noted that we classified known level 1 and 2 actionable “wild type” findings variants for ease of presentation. The most frequent alterations were single nucleotide variants in *TP53* (11.8%), *ATM* (6.1%), and *KRAS* (5.6%), of which 1,496 (61%) were classified as actionable by level 1 (109; 4.4%), level 2 (87; 3.5%), or level 3 (1,300; 52.7%) evidence per our interpretation of FDA guidelines for variant classification (Table 1). Genes with highly actionable variants (level 1 or 2 evidence) were *ALK, BRAF, BRCA1, BRCA2, EGFR, ERBB2, KRAS, MET, NF1, NRAS, ERBB2, KIT, RET*, and *ROS1.* The median number of variants per patient was 3, while the median number of actionable variants (level 1, 2 or 3 evidence) was 2. While it is most common for tests with highly actionable results (level 1 or 2) to have only a single driver mutation identified, there were 18 tests (3%) in this cohort that had 2, and one test with 3 highly actionable findings. This is unlike tests where level 3 (targeted therapy off label or in a trial) is the best CGP option presented, where multiple variants for a given test (in our data, up to 12), may have therapeutic implications. (Supplementary Table 1).

**Table 1:** Interpretation of FDA guidance for actionable variant classification for next generation sequencing

FDA Level of Evidence (LOE)	OmniSeq Comprehensive
**Level 1 (Companion Diagnostic):** Information that is essential for the safe and effective use of a corresponding therapeutic. Supported by analytical validity for the test for each specific biomarker and a clinical study establishing either the link between the result of that test and patient outcomes or clinical concordance to previously approved companion diagnostics.	Variants with evidence of sensitivity, resistance, or non-response indicated as required testing on FDA and/or EMA approved targeted therapy labels for drug administration in the patient’s tumor type.
**Level 2 (Clinically Significant):** Enable health care professionals to use information about their patients’ tumors in accordance with the clinical evidence, such as professional practice guidelines. Supported by demonstration of analytical validity (either on the mutation itself or via a representative approach) and clinical validity based on available clinical evidence.	Variants with evidence of sensitivity, resistance, or non-response to targeted therapeutics based on clinical evidence in professional practice guidelines established by NCCN and/or ESMO for the patient’s tumor type.
**Level 3 (Potential Clinical Significance):** May be informational or used to direct patients towards clinical trials for which they may be eligible. Supported by analytical validation, principally through a representative approach, and clinical or mechanistic rationale for inclusion in the panel, including peer-reviewed publications and *in vitro* pre-clinical models.	Variants that are: (a) can be used to support off-label therapy based on evidence of response in another tumor type, or; (b) therapeutic targets of agents in clinical trials for the tumor type tested

The most common tumor types tested were lung (30.2%), sarcoma (13.1%), colorectal (10.3%), melanoma (7.7%), ovarian (6.9%), prostate (5.0%), breast (4.7%), and uterine (3.2%), with most patients having advanced/metastatic disease (stage III or IV) at the time of test order (84.7%) (Table 2). Treatment naïve patients comprised 139 (22.4%) of tests. Few CGP tests were performed for patients who previously received targeted therapy (2.6%), treatment in a clinical trial (2.6%) or checkpoint blockade immunotherapy (4.4%). Among tests for previously treated patients, 31.9% received 1–2 regimens, 21.5% received 3–5 regimens and 9.5% had received more than 5, with 44.4% of patients actively on drug the day of CGP test order. Several tests were performed for patients with unspecified or unknown prior treatment history (91/620; 14.7%), where medical record data did not provide explicit information (i.e., drug names, exact treatment dates, etc.) about pre-CGP therapies. Average GCP test turnaround time was 8.2 days from the date of specimen receipt.

**Table 2:** CGP test patient characteristics (*n* = 620)

Tumor type tested	Total
Lung	187 (30.2)
Sarcoma	81 (13.1)
Colorectal	64 (10.3)
Melanoma	48 (07.7)
Ovarian	43 (07.7)
Prostate	31 (05.0)
Breast	29 (04.7)
Uterine	20 (03.2)
Neuroendocrine	15 (02.4)
Brain/CNS	14 (02.3)
Esophageal	13 (02.1)
Kidney	10 (01.6)
Pancreas	8 (01.3)
Other Solid Tumor	57 (09.2)
**Tumor stage at the time of test order (number, %)**	
Not Applicable	6 (01.0)
I	8 (01.3)
II	14 (02.3)
III	52 (08.4)
IV	473 (76.3)
Unknown	67 (10.8)
**Prior treatment (number, %)**	
None	139 (22.4)
Chemotherapy/other standard of care	381 (61.5)
Targeted Therapy (variant directed)	16 (02.6)
Immunotherapy (checkpoint blockade)	27 (04.4)
Clinical trial	16 (02.6)
Unspecified	70 (11.3)
Unknown	21 (03.4)
**Number of prior treatment regimens (number, %)**	
0	139 (22.4)
1–2	198 (31.9)
3–5	133 (21.5)
>5	59 (09.5)
Unspecified	70 (11.3)
Unknown	21 (03.4)
**On drug at the time of test order (number, %)**	275 (44.4)
**Turnaround time from specimen receipt (avg. days)**	8.2
**Most actionable variant level of evidence per test**	
Level 1 (companion diagnostic)	95 (15.3)
Level 2 (practice guidelines)	81 (13.1)
Level 3 (off-label/clinical trials)	361 (58.2)
Variants of unknown significance	49 (07.9)
No variants detected	34 (05.5)
**Deceased (as of September 2018)**	318 (51.3)

Unspecified = medical records indicated approximately when patient was last treated but electronic data lacked specific regimen details.

Unknown = medical records did not indicate whether or not patient was previously treated.

On a per-test basis, 176/620 (28.4%) of GCP reports strongly advised physicians about targeted therapy use (indicated or not indicated) based on level 1 companion diagnostic (15.3%) or oncology practice guidelines (13.1%) as the single most actionable finding. The best recommendations provided by the remaining tests advised about the potential of administering targeted therapy off-label or in the investigational setting (level 3 evidence, 58.2%), reported only variants of unknown significance (7.9%), or had no variants detected (5.5%). Patients for over half of all CGP tests (51.3%) were deceased by the end of the study observation period (September 2018).

**Figure 3:**
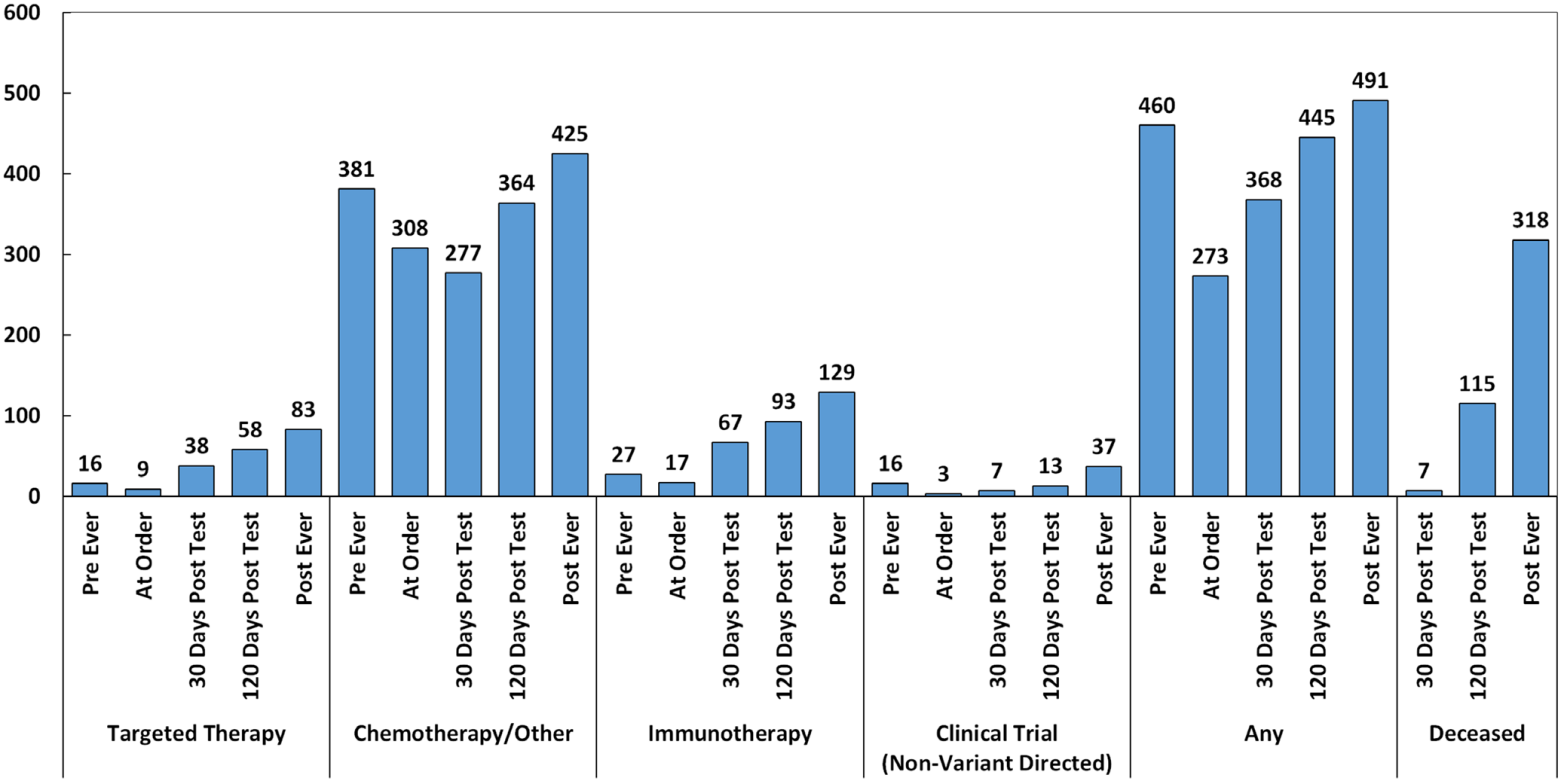
Treatments administered pre-CGP, day of order, post-30 days, post-120 days, and post-ever (*n* = 620). Targeted therapy includes targeted agents administered based on companion diagnostic or practice guideline evidence, off-label, or in clinical trials.

As seen in Figure 3, 83/620 (13.4%) of patients ultimately received targeted therapy (based on companion diagnostic, practice guideline, off-label, or variant directed trial supporting evidence), 129/620 (21%) received immunotherapy (checkpoint blockade), 37/620 (6%) went on non-variant directed clinical trials, and 491/620 (69%) received another standard of care treatment including chemotherapy, radiation, hormone therapy, and non-variant directed targeted agents. Notably, the frequency of chemotherapy/other standard of care treatment was lowest 30 days post-test, unlike other treatment types, where it was lowest the day of the test order.

### Uptake of CGP recommendations by evidence group

To illuminate the extent to which CGP was used by oncologists to make these treatment decisions, we grouped CGP test results the way oncologists consider them—by the strength of the variant association with targeted therapy (level of evidence), variant effect (sensitivity or resistance to targeted therapy), and in the context of patient prior treatment history and status at the time of order (active, previous or treatment naïve). We categorized tests into five (5) mutually exclusive evidence groups based on each test’s single most actionable result: level 1 and 2 targeted therapy sensitivity variants; level 1 and 2 targeted therapy resistance variants; level 3 off label or targeted therapy clinical trial variants; variants of unknown significance, or; no variants detected. For each of these evidence groups, we then compared post-test treatments received to the most actionable test recommendation, in the context of pre-test treatment history.

**Figure 4:**
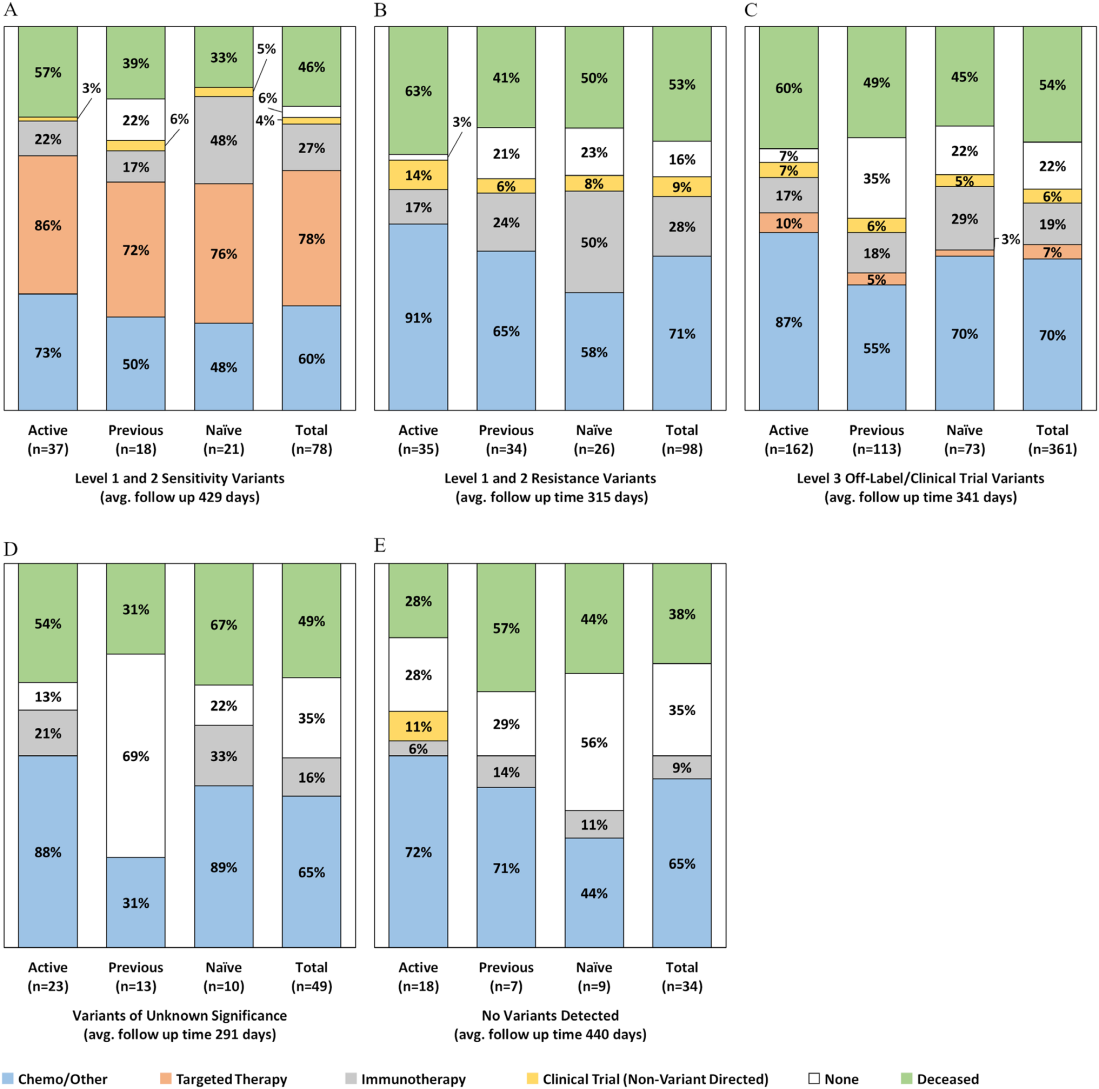
Post-CGP test treatments administered by evidence group and treatment history status at the time of order (*n* = 565). Excludes 69 patients lost to follow up and 21 patients with unknown prior treatment history.

As seen in Figure 4A, there were 78 tests (12.6%) classified as having level 1 or 2 targeted therapy sensitivity variants as the most actionable result, with 61/78 (78%) of the patients in this group receiving a CGP-recommended targeted agent by the end of the observation period. Patients in active treatment at the time of test order had the highest frequency of targeted therapy uptake (32/37; 86%), compared to treatment naïve patients (16/21; 76%) and patients whose previous (last) treatment was administered at least 120 days prior to testing (13/18; 72%). As detailed in Supplementary Table 2, among patients in this evidence group, 68/78 (87%) never received targeted therapy prior to testing, 5 (6%) remained on the same targeted agent they were receiving when the test was ordered, and 5 (9%) eventually received a different targeted agent for the CGP detected alteration. For example, a NSCLC patient on erlotinib for an EGFR L858R at the time of test order had an EGFR T790M variant detected by CGP and subsequently switched to the third-generation EGFR TKI, osimertinib. A breast cancer patient with ERBB2 amplification switched from single agent trastuzumab to combination ado-trastuzumab + pertuzumab.

In this evidence group, nearly half (10/21; 48%) of treatment naïve patients harboring targeted therapy sensitive alterations received immunotherapy by the end of the study period (Figure 4A). Most of these patients however, (6/10) also received targeted therapy (Supplementary Table 2), either before (2/10) or after (4/10) receiving first line checkpoint blockade [data not shown]. One treatment naïve NSCLC patient who underwent targeted therapy prior to immunotherapy had a BRAF V600E mutation detected and received both BRAF single agent and combination BRAF/MEK inhibitors. The second patient who received targeted therapy followed by immunotherapy had KIT mutation in melanoma and received imatinib followed by combination imatinib + pembrolizumab. The patients (5/11) who received immunotherapy despite being eligible for targeted agents comprised four (4) melanoma cases with BRAF V600E mutations and one (1) NSCLC patient with an uncommon EGFR exon 20 insertion (A763_Y764insFQEA), which unlike most other EGFR mutations, is associated with sensitivity to EGFR TKIs.

There were 98 tests (15.8%) where the most actionable variant had strong (level 1 or 2) evidence associated with targeted therapy resistance or lack of benefit (Figure 4B). No patients in this evidence group received variant directed targeted therapy agents. As detailed in Supplementary Table 2, nearly all these tests (90/98; 92%) were either for NSCLC patients harboring *KRAS* mutations indicating they should not receive targeted EGFR tyrosine kinase inhibitors (60/98; 61%), or for colorectal cancer patients harboring *KRAS* and/or *NRAS* mutations (32/98; 33%) indicating they should not receive cetuximab and/or panitumumab. The remaining patients in this group (3/98; 3%) were sarcoma (GIST) patients with *NF1* mutations indicating they should not receive imatinib, or NSCLC patients with EGFR T790M mutations (3/98; 3%) indicating resistance to first and second generation EGFR TKIs. Patients in this evidence group who were in active treatment at the time of test order had the highest use of chemotherapy (32/35; 91%), as well as non-variant directed clinical trial enrollment (5/35; 14%) by the end of the study observation period. Like patients with targeted therapy sensitivity variants, half of treatment naïve patients with resistance variants (13/26; 50%) received checkpoint blockade immunotherapy by the end of the observation period.

More than half of the CGP tests in this analysis (361/620; 58%) harbored level 3 alterations as their most actionable targeted therapy recommendation, indicating these patients may respond to targeted therapy in the off-label or investigational setting (Figure 4C). In this evidence group, CGP detected more than 1,000 variants associated with inclusion criteria or direct targets for therapies in clinical trials, and reported more than 200 variant-specific off-label targeted therapy recommendations. Overall, patients for 24 tests (7%) in this evidence category received targeted therapy, and as detailed in Supplementary Table 2, 13 (4%) patients received off-label targeted therapy and 11 (3%) patients received targeted therapy in a variant-directed clinical trial, predominantly PARP inhibitors and BRAF or BRAF/MEK inhibitors. We again observed that treatment naive patients in this group were more likely to elect immunotherapy (29%) compared to patients who were previously treated (18%) or in active treatment (17%) at the time of order. On average, 70% of patients in the level 3 evidence group remained on, or initiated standard of care chemotherapy, radiation, hormone therapy, or other non-variant directed targeted therapy, particularly patients already in active treatment at the time of test order (87%). Previously treated patients in this group also had the greatest number of patients (40/110; 35%) who received no further treatment by the end of the study period.

Patient tests with only variants of unknown significance detected (*n* = 49/620; 8%) had a high proportion of patients (35%) who ultimately received no therapy, particularly previously treated patients (69%) (Figure 4D). No patients in this group received off-label targeted therapy or enrolled in any clinical trials, variant directed or otherwise. Similarly, patients with no variants detected at all (*n* = 34/620; 5%), mostly received either chemo or other standard treatments (65%), if anything, with some clinical trial enrollment (11%) among active treatment patients. Overall, this group also had the lowest number of patients undergo immunotherapy and the lowest percentage of patients deceased at the last date of follow up.

**Figure 5:**
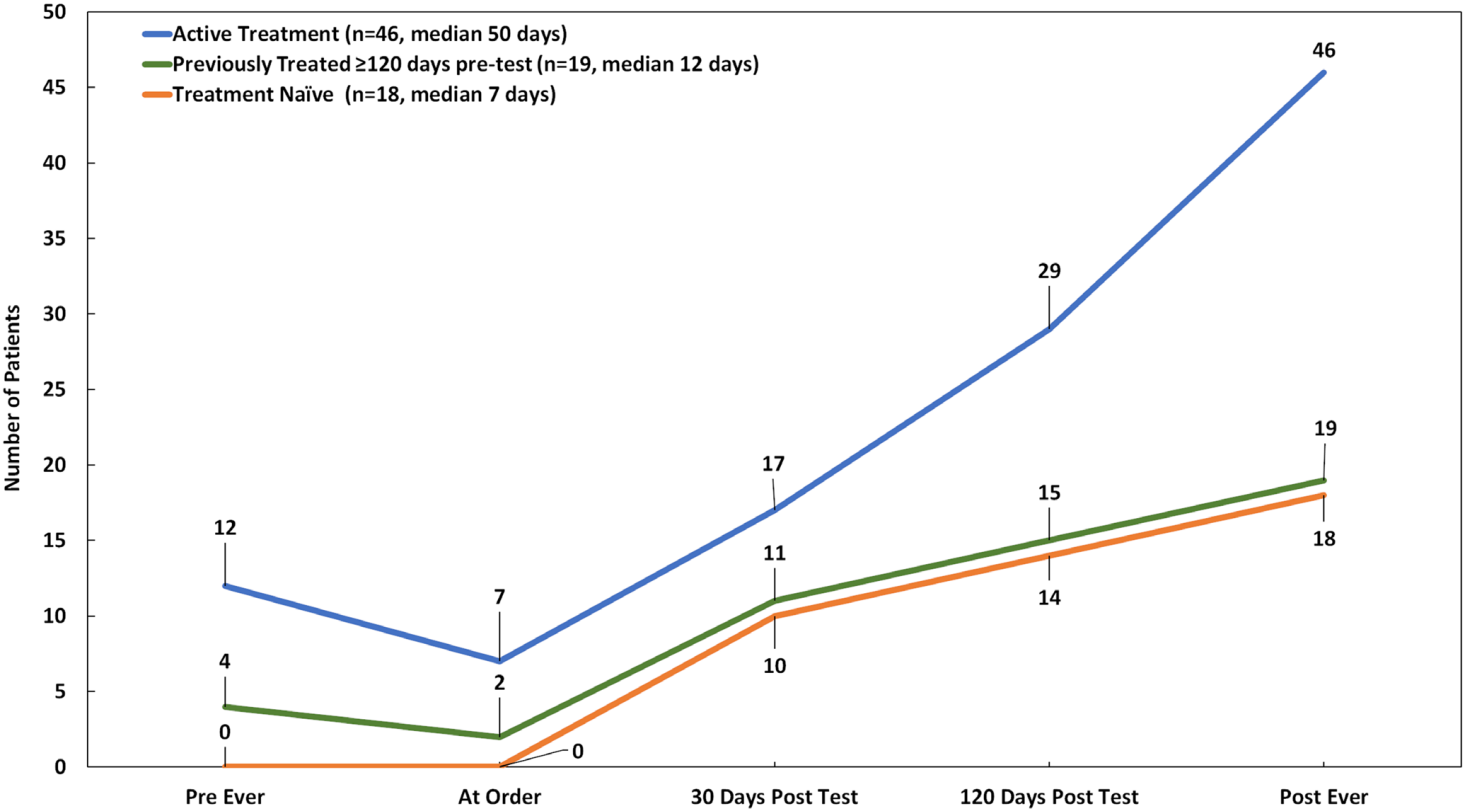
Targeted therapy uptake over time by treatment history status at the time of order (*n =* 83). Targeted therapy includes targeted agents administered based on companion diagnostic or practice guideline evidence, off-label, or in clinical trials.

Given that we observed post-test targeted therapy use was most common among patients who were already actively receiving treatment when CGP was ordered, and that few patients were on targeted therapy at the time of test order, we examined how long it took to start targeted therapy. As seen in Figure 5, while patients who went on targeted therapy had similar average follow up time regardless of prior history, median time from CGP results to initiation of targeted therapy was much longer for patients in active treatment at the time of test order (50 days), compared to their previously treated (12 days) or treatment naïve (7 days) counterparts, despite eventually initiating targeted therapy for twice as many patients (*n* = 46/83; 55.4%).

## DISCUSSION

The variety of CGP testing applications for targeted therapy across many tumor types and lines of treatment in standard practice requires knowledge about patient history and current treatment needs to determine test clinical utility. The information asymmetry that exists between oncologists, who have deep first-hand knowledge about their patients, and the reference laboratories that receive very little of this information when CGP tests are ordered, creates a significant challenge to assessing utility. While it is not possible for testing laboratories to fully know clinician intent regarding therapeutic selection or modification, government and private payers require comprehensive genomic profiling be medically necessary for patient treatment to be covered as a service under health plans. Additionally, to comply with insurance prior authorization requirements, physicians must submit medical documentation evidencing clinical need.

We uniquely address the deficit in understanding CGP utility in standard practice by using real world medical record data to assess the impact of treatment history status at the time of CGP test order (naïve, active, or previously treated) as a proxy measure of oncologists’ intended use of results (treatment for first line, progression, or recurrence, respectively) on targeted therapy decision making. We excluded patients with inadequate information from analysis of treatment decisions as lost to follow up, a common scenario at comprehensive cancer centers that perform second opinions. We completed chart review to confirm treatment history status, a critical variable in the study. We then contextually assessed treatment decisions from the viewpoint of oncologists, based on strength of therapeutic variant association, supporting clinical evidence, variant effect (sensitivity versus resistance), and patient treatment history.

Tested patients in our study were representative of the solid tumor population for which approved, beneficial targeted treatment options are available, with advanced/metastatic lung, colorectal, melanoma, sarcoma, ovarian and breast cancers making up over 75% of cases. We found however, that the tested population was pre-treated, with >30% receiving 3 or more regimens prior to testing, and 44% actively receiving therapy at the time of test order (1% variant-directed targeted therapy). While CGP results with strong clinical evidence supporting targeted therapy led to use of targeted agents the clear majority of the time (86%), it also took a median of 50 days to initiate targeted treatment, compared to 7 days for treatment naïve patients, and 12 days for previously treated patients. Taken together, these findings highlight the possibility that many patients, who are likely to benefit from targeted therapy, could be tested sooner in their treatment journey, prior to initiating other standard therapies.

Because CGP assesses all “shots on goal” for targeted therapy by testing all major variant types in cancer associated genes at one time, coupled with the fact that highly actionable targeted therapy sensitivity variants are somewhat uncommon (13% in this study), CGP testing inherently has negative predictive value. Our analysis confirms this, as CGP appears to frequently be used to appropriately rule out targeted therapy and inform patients’ next best option, including chemotherapy, palliative care, or hospice. While the most common example of this in our data is *KRAS* mutations in colorectal (lack of benefit for cetuximab) and lung cancer (lack of benefit for EGFR inhibitors), there is also growing evidence to support testing for acquired resistance mutations in *ALK* for example, where clinical benefit from newer second and third generation *ALK* inhibitors for NSCLC differs based on the specific mutation identified [28].

Overall, 22% of patients tested chose checkpoint inhibitors, and immunotherapy use was particularly high among treatment naïve patients, including 48% of patients who were also eligible for targeted therapy. Thus, our analysis underscores the immediate and growing need for simultaneous comprehensive genomic and immune profiling for standard of care treatment decision making. Additional targeted therapy and immunotherapy approvals and breakthrough designations singularly make comprehensive response marker testing across both treatment modalities, as well as the provisioning of associated comparative efficacy data from clinical studies, critical supporting oncologists’ treatment decision making.

The limited use of CGP for off-label treatment or enrollment in clinical trials is unsurprising, and has been widely reported by others. Achieving the critical mass needed to demonstrate clinical benefit for hundreds of variant-disease-drug associations within a single institution trial is still a challenge due low frequency of actionable variants and well-known barriers resulting in universally low clinical trial enrollment [29, 30]. In a recent follow-on analyses, the MD Anderson IMPACT precision medicine initiative, arguably the biggest and longest running precision medicine program in the United States, reported positive outcomes from matched targeted therapies results for only 1,436 patients after a full 10 years of operation [14]. Our study highlights the same finding of low targeted therapy off-label use and clinical trial enrollment in the standard of care setting.

Our findings support that in real world practice, oncologists utilize CGP results consistently and contextually, based on anticipated patient treatment needs at the time of order and the assay’s most actionable genomic finding to rule targeted therapy in or out. Importantly, this work highlights the use of CGP results as a longitudinal treatment planning tool for patients who received other standard of care treatments prior to CGP testing.

## MATERIALS AND METHODS

CGP testing was performed in a CLIA certified laboratory at OmniSeq, Inc. (Buffalo, NY, USA), using OmniSeq Comprehensive^®^, a commercially available test approved for clinical use by the New York State Clinical Laboratory Evaluation Program (NYS CLEP). OmniSeq Comprehensive uses tumor tissue to identify all classes of somatic genomic alterations in 144 cancer-associated genes. Specifically, the DNA-Seq component of the test detects single nucleotide variants, insertions, deletions, indels, and copy number variants while the RNA-Seq component performs rearrangement (fusion) analysis in oncogenes. DNA mutational analysis also detects loss-of-function mutations in tumor suppressor genes using a complete coding sequence coverage strategy, while copy number analysis detects homozygous deletions. The test’s bioinformatics pipeline filters single nucleotide polymorphisms and identifies reportable variants, including variants of unknown significance (VUS), based on pathogenicity using multiple public genomic content sources such as COSMIC, 1000 Genomes Project, dbSNP, SIFT, PolyPhen, and ClinVar.

OmniSeq Comprehensive does not sequence matching non-tumor tissue from tested patients, however, germline mutations can be identified from tumor-only sequencing results without direct analysis of germline DNA. As such, the test reports detected mutations in genes prescribed by the American College of Medical Genetics and Genomics (ACMG) [31] as potentially hereditary, and directs physicians to further investigate by germline testing if clinically applicable.

The test was designed to require low DNA and RNA sample inputs requirements (1ng-30ng) by using proprietary methods to extract nucleic acids from clinical formalin fixed paraffin embedded (FFPE) tissues samples that have minimal tumor cells present. Of note, while similar profiling assays have reported DNA yield failure rates of 4.9% [32] and 6.0% [33], the DNA yield failure rate for OmniSeq Comprehensive was only 0.5% in this study primarily due to technological advances in sequencing [34, 35] and improved methods of DNA isolation [36].

OmniSeq Comprehensive test performance characteristics were analytically validated by OmniSeq Laboratories under the requirements of the Clinical Laboratory Improvement Amendments (CLIA) of 1988, and OmniSeq, Inc. is licensed by CLIA, College of American Pathologists (CAP), and the NYS CLEP to perform high-complexity molecular diagnostic testing. Additional details regarding OCP methodology, clinical validity, and performance characteristics can be found in the National Center for Biotechnology Information (NCBI) Genetic Testing Registry (https://www.ncbi.nlm.nih.gov/gtr/tests/552042/overview/).

Roswell Park Comprehensive Cancer Center clinicians ordered 777 CGP tests between June 2016 and June 2017 as part of usual patient care (Figure 1). We excluded from analysis, orders that were never completed due to clinician cancelation (*n* = 36; 4.6%), failed tissue QC (*n* = 44; 5.6%), nucleic acid quantity not sufficient (QNS) (*n* = 4; 0.5%), and tests for patients lost to follow up due to death within 30 days of report with no subsequent treatment (*n* = 38; 4.9%) and tests for patients who had not subsequent visits or therapy within 30 days of CGP report (*n* = 35; 4.5%).

OmniSeq Comprehensive reports therapeutic variant associations for Food and Drug Administration (FDA) and European Medicines Association (EMA) approved targeted therapies, National Comprehensive Cancer Center (NCCN) and European Society for Medical Oncology (ESMO) professional practice guidelines, and as captured in inclusion/exclusion criteria for clinical trials based on data from www.clinicaltrials.gov. For this study, each reported variant was mapped to one or more levels of evidence based on interpretation of FDA guidance for actionable variant classification for next generation sequencing [27] (Table 1). The most actionable targeted therapy recommendation was then determined for each CGP test (with level 1 companion diagnostic evidence being the strongest). For example, in a NSCLC report with *KRAS* G12C detected, the *KRAS* mutation may be associated with inclusion or exclusion criteria in clinical trials regardless of the associated therapy (level 3), but its most actionable report-wide association is resistance to EGFR inhibitors based on clinical practice guidelines (level 2). We asserted 4 evidence groups for CGP results based on how oncologists use them: level 1 and 2 targeted therapy sensitivity variants; level 1 and 2 targeted therapy resistance variants; level 3 off-label/clinical trial variants; and variants of unknown significance.

Electronic pharmacy records as of September 2018 were used to classify treatments received by tested patients as targeted therapy (variant-directed) regardless of setting (i.e., standard of care, off-label, or investigational), immunotherapy (i.e., anti-PD-1 checkpoint inhibitor), non-variant directed clinical trials, or other standard therapy (chemotherapy, non-variant directed targeted therapy, radiation, hormone therapy, or other therapy). Patient treatment history status at the time of CGP test order was classified as: treatment naïve (no prior history of systemic therapy); active (currently on systemic therapy), or; previously treated (most recent systemic therapy ≥ 120 days prior to test order), and was manually verified by chart review. Electronic pharmacy records were used to determine whether each treatment occurred either pre-CGP order, day of CGP order, 120 days post-CGP test results, or ever (by the end of the study period or death), as well as number of regimens prior to testing, up to and including the order date. Follow up time was calculated from the date of CGP report to the last patient visit or date of death as of September 2018, if applicable. We excluded patients from treatment decision analysis if they were considered lost to follow up, including patients who died <30 days of results with no subsequent treatment (*n* = 36), and patients with <30 days follow up with no subsequent visits to Roswell Park (*n* = 33). For each report, we then compared and described the frequencies of targeted therapy and other treatment(s) received by patients through the end of the observation period by results evidence group and patient treatment history status.

## SUPPLEMENTARY MATERIALS






