# Supplementary material for: Oncologist uptake of comprehensive genomic profile guided targeted therapy
Source: Oncotarget. 2019 Jul 23;10(45):4616–29. doi: 10.18632/oncotarget.27047 (PMC6659802; doi:10.18632/oncotarget.27047)
Supplement: Supplementary file 1 [file oncotarget-10-4616-s001.pdf]

Oncologist uptake of comprehensive genomic profile guided targeted therapy

SUPPLEMENTARY MATERIALS

Supplementary Table 1: Number of variants per test by level of evidence for therapeutic association(s) (*n* = 620). See Supplementary_Table_1

Supplementary Table 2: CGP guidance and targeted therapy decisions by evidence group and treatment history status: Tests with variants supported by level 1 or level 2 evidence (*n* = 176), and tests with level 3 variants resulting in targeted therapy given off-label or in a clinical trial (*n* = 24). See Supplementary_Table_2
